# Modifications of Glucocorticoid Receptors mRNA Expression in the Hypothalamic-Pituitary-Adrenal Axis in Response to Early-life Stress in Female Japanese Quail

**DOI:** 10.1111/jne.12228

**Published:** 2014-11-17

**Authors:** C Zimmer, K A Spencer

**Affiliations:** School of Psychology and Neuroscience, University of St AndrewsSt Andrews, UK

**Keywords:** prenatal stress, postnatal stress, mineralocorticoid receptor, glucocorticoid receptor, hypothalamic-pituitary-adrenal axis programming

## Abstract

Stress exposure during early-life development can programme individual brain and physiology. The hypothalamic-pituitary-adrenal (HPA) axis is one of the primary targets of this programming, which is generally associated with a hyperactive HPA axis, indicative of a reduced negative-feedback. This reduced feedback efficiency usually results from a reduced level of the glucocorticoid receptor (GR) and/or the mineralocorticoid receptor (MR) within the HPA axis. However, a few studies have shown that early-life stress exposure results in an attenuated physiological stress response, suggesting an enhance feedback efficiency. In the present study, we aimed to determine whether early-life stress had long-term consequences on GR and MR levels in quail and whether the effects on the physiological response to acute stress observed in prenatally stressed individuals were underpinned by changes in GR and/or MR levels in one or more HPA axis components. We determined GR and MR mRNA expression in the hippocampus, hypothalamus and pituitary gland in quail exposed to elevated corticosterone during prenatal development, postnatal development, or both, and in control individuals exposed to none of the stressors. We showed that prenatal stress increased the GR:MR ratio in the hippocampus, GR and MR expression in the hypothalamus and GR expression in the pituitary gland. Postnatal stress resulted in a reduced MR expression in the hippocampus. Both early-life treatments permanently affected the expression of both receptor types in HPA axis regions. The effects of prenatal stress are in accordance with a more efficient negative-feedback within the HPA axis and thus can explain the attenuated stress response observed in these birds. Therefore, these changes in receptor density or number as a consequence of early-life stress exposure might be the mechanism that allows an adaptive response to later-life stressful conditions.

Stress exposure during early life has long lasting impacts on both the structure and function of several tissues associated with a higher risk of developing health pathologies and behavioural disorders 1–4. The concept of developmental programming (i.e. permanent changes in physiology and neural systems following early-life experiences) has been suggested to explain the negative consequences of environmental stress exposure during early development. During this programming, environmental adversity experienced by the mother initiates maternal responses, which in turn affect the development of her offspring, including the organisation and functioning of specific tissues, especially the brain 2–4. The main focus of research on these organisational effects has been their role in facilitating higher risk to later diseases or syndromes (e.g. cardiovascular disease, depression). However, the environmental matching hypothesis proposes that developmental programming may prime the offspring to cope better with stressful conditions and thus may be adaptive when environmental conditions in later life match those experienced during early stages. The negative consequences of early-life adversity may therefore simply result from a mismatch between conditions between environmental conditions at different life stages 5–7.

One fundamental physiological system that links an individual to changes in its environment is the hypothalamic-pituitary-adrenal (HPA) or stress axis 1,7,8. This axis is activated during in both development and adulthood when a stressor is perceived in the brain ultimately resulting in the release of glucocorticoid hormones from the adrenal cortex. The increase in glucocorticoid levels facilitates a switch of physiological processes and behaviours from non-essential activities to those that promote short-term survival, such as increased locomotion and mobilisation of energy stores. This response is tightly regulated by a negative-feedback loop at the level of the hippocampus, hypothalamus and anterior pituitary to shut the HPA axis down and to return to a homeostatic point, avoiding the negative consequences of chronically elevated glucocorticoids 3,9. The effects of glucocorticoids in the brain, as well as the tight regulation of the axis, are mediated by two intracellular receptors: the mineralocorticoid receptor (MR) and the glucocorticoid receptor (GR) 10–12. In both mammals and birds, GR occur everywhere in the brain but are most abundant in the hippocampus and hypothalamus and are also found in the pituitary gland. MR are mostly found in the hippocampus, and also in the hypothalamus, and bind glucocorticoids with a five- to ten-fold higher affinity than GR 11–15. Consequently, MR remain activated during periods of basal secretion and are involved in the maintenance of integrity and stability of the HPA axis, primarily determining stress sensitivity of this axis. MR can also boost the initial acute stress response and promote behavioural adaptation to stress during novel situations 11,12,16,17. GR are additionally recruited when the glucocorticoid levels rise further, preventing the initial reaction from overshooting by bringing back cells to baseline levels via inhibition of the HPA axis. They also facilitate the recovery from stress and are involved in the mediation of memory consolidation by glucocorticoids 10–12,16. Because both MR and GR are involved in the regulation of the stress response and in the negative-feedback loop, it has been proposed that the GR:MR balance is crucial to maintain homeostasis and its disruption may compromise stress resilience and affect behaviour 11–13,15–17.

The HPA axis is one of the primary targets of early-life stress programming in the brain 1,2,4,8,18. High glucocorticoid levels during early development can permanently alter HPA axis functioning via alteration of baseline or stress-induced glucocorticoid levels and/or negative-feedback during stress recovery. It has been generally shown that an increase in pre- or postnatal stress programmes a hyperactive HPA axis, indicative of a reduced negative-feedback 1–4,8,19–22. However, a few studies have shown that early-life stress exposure can result in an attenuated physiological stress response later in life, suggesting enhanced negative-feedback efficiency 23–26. This programming of the HPA axis has been ascribed to modifications of GR and/or MR expression in the hippocampus and other feedback sites 1–3,18. It has been recently shown in the Japanese quail (*Coturnix japonica*) that exposure to early-life stress can permanently program physiology and behaviour in a potentially adaptive way 26. At the physiological level, prenatal stress altered the HPA axis functioning in a way that attenuated the acute stress response suggesting an increased negative-feedback efficiency within the HPA axis 26.

In the present study, we aimed to determine whether early-life stress had long-term effects on GR and MR levels and whether the effects on the physiological response to acute stress observed in prenatally stressed individuals were underpinned by permanent modifications of the level of both glucocorticoids receptors (MR and GR) in feedback sites within the HPA axis. In the same quail, as used previously 26, we measured the relative mRNA expression of MR and GR in the hippocampus, hypothalamus and pituitary gland using a quantitative real-time polymerase chain reaction (PCR). Because prenatal stress resulted in an attenuated stress response, we predicted that GR expression should be enhanced in the hippocampus, hypothalamus and pituitary gland, resulting in a more efficient negative-feedback in prenatally stressed quail. In the hippocampus, the balance between GR and MR is crucial for resilience from a stressor and an increase in GR level or a decrease in MR level in the hippocampus could lead to more pronounced effects of GR, facilitating the recovery from stress 10–12,17. We consequently hypothesised that the GR:MR ratio should be reduced in the hippocampus of quail exposed to prenatal stress.

## Materials and methods

### Experimental manipulations

Prenatal stress was manipulated by injecting eggs with 10 μl of corticosterone (CORT) (concentration: 850 ng/ml; Sigma Aldrich, Poole, UK) dissolved in sterile peanut oil at the egg apex under sterile conditions on day 5 of incubation (B). This increased the endogenous CORT concentration in the yolk within 1.8 SD above control yolks, which was determined by radioimmunoassay and liquid chromatography-mass spectroscopy and is similar to previous studies that have increased CORT levels within physiologically relevant ranges 24,27. Control eggs (C) were injected with peanut oil alone. Chicks of each prenatal treatment were subsequently randomly allocated to one of two postnatal food treatments: either food removal for 25% of daylight hours (3.5 h) on a random daily schedule for 15 days from post-hatching day 4 (F−) or *ad lib*. food at all times (C). Random removal of food has been shown to increase stress hormones in birds, without causing food restriction 28,29. After this postnatal treatment, all birds had access to *ad lib*. food (Standard Layer Pellet, BOCM, Wherstead, UK) 26,30. The present study was part of a large experiment looking at the long-term and trans-generational effects of pre- and/or postnatal stress exposure in the Japanese quail. For GR and MR expression in this F1 generation, we focused on females because one of the aims of the project was to look how the maternal developmental environment affects offspring phenotype. We thus had four treatment groups: prenatal control/postnatal control (CC; n = 6); prenatal control/postnatal food- (CF−; n =* *6), prenatal CORT/postnatal control (BC; n =* *12); and prenatal CORT/postnatal food- (BF−; n =* *6). All experimental procedures were carried out under Home Office Animals (Scientific) Procedures Act project licence 60/4068 and personal licence 70/1364 and 60/13261.

### Tissue collection and quantitative real-time PCR

At the end of the experiment, when females were 246.5 ± 1.4 (SEM) days old, they were sacrificed by injection of an overdose of Dolethal (Vetoquinol, Buckingham, UK). Brains were quickly removed (within 1 min) then pituitary glands that lie on the underside of the brain in the centre of the floor of the cranium were also removed using forceps (within 1 min after brain removal) and placed on dry ice until frozen, then stored at −80 °C. To perform the dissections, the brains were placed ventral side up into a brain matrix (Roboz Surgical Instrument Co., Gaithersburg, MD, USA) with a 1-mm graduated scale placed on a mixture of dry and wet ice to keep the brain frozen and a 2-mm thick coronal section was cut using two razor blades positioned approximately 4 mm from the rostral pole and 2 mm from the cerebellum. The plane of cutting was adjusted to match as closely as possible the plane of the chicken brain atlas 31. Then, when still frozen, two equivalent bilateral punches (1 mm in diameter each) were obtained from the hippocampus and a single punch was obtained from the medial hypothalamus that spanned the third ventricle. Each sample was stored separately at −80 °C.

Total RNA was extracted and purified using Absolutely RNA Miniprep kits (Agilent Technologies, Santa Clara, CA, USA) in accordance with the manufacturer's instructions. The quantity and integrity of RNA were assessed with a RNA 6000 Pico assay kit for hippocampus and hypothalamus and a RNA 6000 Nano kit for pituitary gland using the Agilent 2100 Bioanalyzer (Agilent Technologies) in accordance with the manufacturer's instructions. The mean RIN number of these samples is 8.2 ± 0.1 (range 5.2–10). First-strand cDNA was synthesised using Affinity Script Multiple Temperature cDNA Synthesis kits (Agilent Technologies) and diluted to obtain a final concentration of 25 pg/μl. This resulting cDNA was used to perform quantitative real-time PCR (qPCR) for the genes of interest [GR and MR and the house-keeping gene β-actin (BA)] for the different brain regions using gene-specific primers. BA was determined as the best candidate house-keeping gene for our samples (M =* *0.30, other candidates M ≥ 0.34) using a chicken (*Gallus gallus*) GeNorm kit (Primerdesign, Southampton, UK). Specific PerfectProbe™ primers (Primerdesign) were designed based on published chicken nucleotide gene sequences and were validated using quail cDNA by Primerdesign. These primers amplified single products with no dimer pairs. GR sense primer: TAATGACCGTGGTGACCTTTTA, anti-sense primer: TTTCTTGCTTTATGCCAGGAGTA (GenBank accession number NM_001037826). MR sense primer: GTAGAATAGAGGACAGATGAACTTTT, anti-sense primer: ACCCAGAGAGAACACTACAGAT (GenBank accession number NM_001159345). All qPCR reactions were run in duplicate and were performed in 20-μl reactions containing 10 μl of 2 × Brilliant III Ultra-Fast QPCR Master Mix (Agilent technologies), 1 μl of specific PerfectProbe™ primer (Primerdesign) at a working concentration of 300 nm, 0.3 μl of reference dye, 3.7 μl of RNAse/DNAase-free water and 5 μl of appropriate cDNA along with no-template controls and blanks. Reactions were carried out on a Stratagene MX 3005P (Agilent Technologies) at 95 °C for 3 min, then 50 cycles of 95 °C for 20 s and 60 °C for 20 s. From standard curves generated with known concentration of cDNA, we determined that the amplification efficiency [Eff =* *10^(−1/slope)^−1] was higher than 95% for GR, MR and BA. Therefore, we used the delta C_t_ method (ΔC_t_) to quantify the relative expression of GR and MR relative to BA: 2^−(C^t ^GR/MR – Ct BA)^ 32.

### Statistical analysis

To compare the relative expression of both receptors in the different regions of the HPA axis, we used a generalised linear model (GLM) fitted with a gamma law because the residuals of linear models were not normally distributed, using the genmod procedure in sas, version 9.4 (SAS Institute Inc., Cary, NC, USA). Receptor type (GR or MR), tissues (hippocampus, hypothalamus and pituitary gland) and their interaction were used as fixed factors. To determine the consequences of exposure to early-life stress on the GR:MR ratio, MR and GR relative expression, we also used GLMs fitted with a gamma law because the residuals of linear models were not normally distributed. Pre- and postnatal treatments and their interactions were specified as fixed factors. For multiple comparisons, Tukey–Kramer adjustment was applied to obtain a corrected value. P < 0.05 was considered statistically significant. Data are presented as the mean ± SEM.

## Results

### GR and MR mRNA expression in the different HPA axis regions

**Fig 1:**
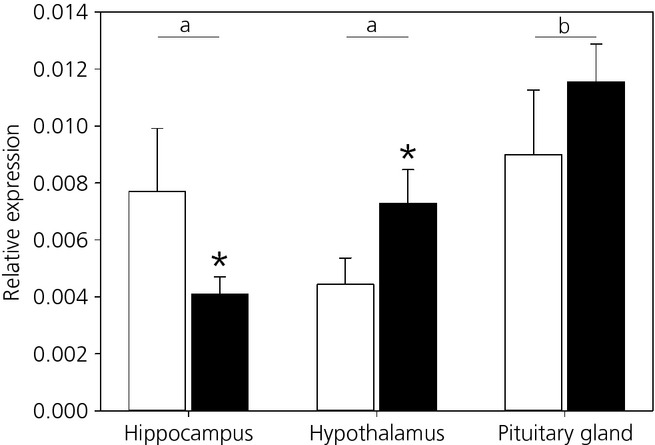
Mean ± SEM relative gene expression of the mineralocorticoid receptor (white) and glucocorticoid receptor (black) in the hippocampus, hypothalamus and pituitary gland in all females. Different lowercase letters indicate a significant difference between tissues. *Statistically significant difference within tissues.

The expression of both receptors (MR and GR) was different between the three HPA axis regions (χ²_ _=* *15.80, d.f. = 1,189, P =* *0.0004) (Fig. 1), with higher expression in the pituitary gland compared to both other tissues (Z> 3.39, P < 0.002). The expression of both receptors was different within each tissue (receptor × tissue: χ²_ _=* *11.71,, d.f. = 1,189, P =* *0.003) (Fig. 1). In the hippocampus, MR was more highly expressed than GR (Z =* *2.58, P =* *0.01). In the hypothalamus, GR was highly expressed compared to MR (Z =* *2.01, P =* *0.04). Finally, in the pituitary gland, MR and GR relative expression did not differ (Z =* *1.04, P =* *0.30).

### Effects of early-life stresses on receptor mRNA expression

#### Hippocampus

**Fig 2:**
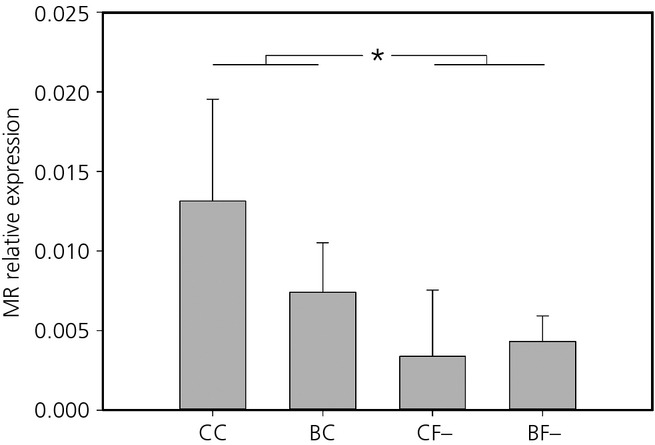
Mean ± SEM relative expression of the mineralocorticoid receptor (MR) in the hippocampus of quail in the four treatment groups: pre-hatching and post-hatching control (CC), pre-hatching control and post-hatching unpredictable food availability (CF−), pre-hatching corticosterone-treated and post-hatching control (BC) and both treatments (BF−). *Statistically significant difference.

**Fig 3:**
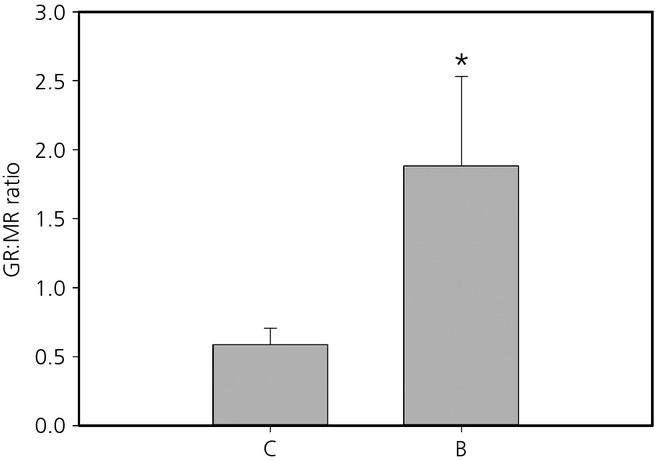
Mean ± SEM glucocorticoid receptor (GR):mineralocorticoid receptor (MR) ratio in the hippocampus of prenatal control (C) and prenatally stressed (B) quail. *Statistically significant difference.

Mineralocorticoid receptor relative mRNA expression in the hippocampus was significantly reduced in postnatally stressed compared to postnatal control quail (χ²_ _=* *6.11, d.f. = 1,23, P =* *0.013). Moreover, MR relative expression was significantly influenced by the interaction between both early-life treatments (prenatal treatment × postnatal treatment: χ^2^_ _=* *4.10 d.f. = 1,23, P =* *0.0428). Post-hoc comparisons were not significant; however, it appears that MR expression was the highest in control individuals exposed to none of the stresses (CC) compared to individuals exposed to one or both early stresses (Fig. 2). GR mRNA relative expression in the hippocampus was not affected by any of early-life stresses (Table 1). The GR:MR ratio was significantly affected by early-life stress, with a lower ratio in prenatal control quail compared to prenatally stressed individuals (χ²_ _=* *9.60, d.f. = 1,27, P =* *0.002) (Fig. 3).

**Table 1:** Statistical Results for the Effects of Prenatal Stress, Postnatal Stress Treatments and Their Interaction of the Generalised Linear Models for Mineralocorticoid Receptor (MR) and Glucocorticoid Receptor (GR) Relative Gene Expression in the Hippocampus, Hypothalamus and Pituitary Gland. Bold values are significant effects

	Treatment	MR			GR		
		χ²	d.f.	P-value	χ²	d.f.	P-value
Hippocampus	Prenatal	0.01	1,23	0.925	0.78	1,32	0.378
		Postnatal	6.11	1,23	**0.013**	0.14	1,32	0.711
		Interaction	4.10	1,23	**0.043**	0.51	1,32	0.477
Hypothalamus	Prenatal	4.53	1,30	**0.033**	0.08	1,31	0.781
		Postnatal	0.00	1,30	0.976	1.65	1,31	0.198
		Interaction	2.89	1,30	0.089	5.02	1,31	**0.025**
Pituitary gland	Prenatal	0.09	1,31	0.770	6.16	1,24	**0.013**
		Postnatal	0.04	1,31	0.838	0.19	1,24	0.660
		Interaction	0.35	1,31	0.553	0.60	1,24	0.437

Bold values are significant effects.

#### Hypothalamus

**Fig 4:**
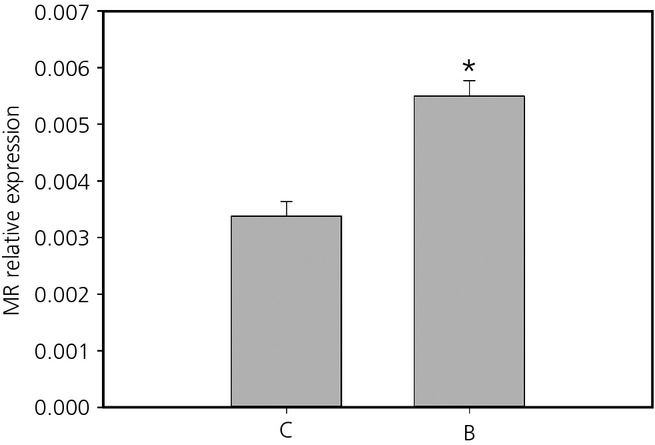
Mean ± SEM relative expression of the mineralocorticoid receptor (MR) in the hypothalamus of prenatal control (C) and prenatally stressed (B) quail. *Statistically significant difference.

**Fig 5:**
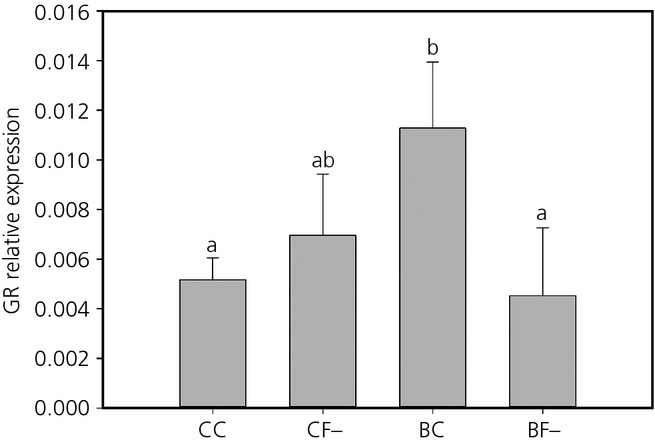
Mean ± SEM relative expression of the glucocorticoid receptor (GR) in the hypothalamus of quail in the four treatment group: pre-hatching and post-hatching control (CC), pre-hatching control and post-hatching unpredictable food availability (CF−), pre-hatching corticosterone (CORT)-treated and post-hatching control (BC) and both treatments (BF−). Different lowercase letters indicate significant differences.

In the hypothalamus, relative MR mRNA expression was increased following exposure to prenatal stress (χ²_ _=* *4.53, d.f. = 1,30, P =* *0.033) (Fig. 4), although there were no effects of postnatal treatments or any interaction between the two stages (Table 1). There were no main effects of either early-life treatment on GR expression, although there was an effect of the interactions between both treatments (χ²_ _=* *5.02, d.f. = 1,31, P =* *0.025). GR expression was higher in individuals only exposed to prenatal stress (BC) than in individuals exposed to none (CC) or both stresses (BF−) (Z > 2.05, P < 0.041) (Fig. 5).

#### Pituitary gland

In the pituitary gland, GR relative expression was significantly higher in quail prenatally exposed to CORT (B: 0.014 ± 0.0022) compared to prenatal controls (C: 0.0093 ± 0.0012) (χ²_ _=* *6.16, d.f. = 1,24, P =* *0.013). There were no effects of postnatal treatment or of the interaction between both treatments (Table 1). There were also no effects of either of our treatments on MR expression (Table 1).

## Discussion

In the present study, we showed that developmental stress had long-term effects on GR and MR receptor mRNA expression in the HPA axis in birds. These permanent modifications of MR and GR expression in the HPA axis are in agreement with a more efficient negative-feedback and could explain the attenuated physiological stress response observed in prenatally stressed quail 26.

Both receptor types were found in the hippocampus, hypothalamus and pituitary gland, as expected from previous work in birds 13–15,19,33. In the hippocampus, MR was highly expressed compared to GR, whereas it was the opposite in the hypothalamus. In the pituitary gland, both receptors were expressed at the same level. This pattern of relative expression of both MR and GR is in agreement with the expression pattern usually observed in birds and mammals 10–15,19,33.

Our early-life treatments had long-term consequences on both receptors expression levels, although these effects were region specific. In addition, prenatal stress had the strongest long-term programming effects on the HPA axis. In the hippocampus, prenatal stress exposure was associated with a higher GR:MR ratio and MR expression was reduced in postnatally stressed quail compared to controls. By contrast to our hypothesis, GR expression was not modified in the hippocampus. Consequently, it appears that the change in the GR:MR ratio in prenatally stressed quail resulted from the effects of early-life stress on MR expression. In the hypothalamus, both MR and GR were affected by prenatal exposure to CORT; however, in the pituitary, prenatal stress only affected GR. Previous work has suggested that the prenatal period is a time of significant sensitivity to stress 2,12,18. However, the effects of early-life stress on GR and MR mRNA levels have produced mixed results. In mammals, prenatal stress usually results in an increased basal or stress-induced glucocorticoid levels associated with a reduced GR and/or MR expression. By contrast, prenatal stress can also be associated with a reduced HPA axis activity as a result of a higher GR expression 1,18,34. For example, in the guinea pig (*Cavia porcellus*), female offspring from mothers exposed to stress on gestational day 60 showed a reduced adrenal reactivity to an acute stressor compare to controls, which was related to the higher GR mRNA level in the hypothalamus measured in these females 35. It has been proposed that the variability of the results of prenatal stress in mammals could be a result of the difference in the timing of stress exposure relative to birth and the nature of the prenatal stress 35. In the chicken, it has been shown that prenatal stress via CORT injection in the eggs resulted in a decrease in GR protein in the hypothalamus in adulthood but GR mRNA levels were not affected 36. In the Japanese quail, stress associated with food restriction between days 4 and 6 after hatching had no effects on GR mRNA expression in the hippocampus but MR expression was not determined 37. In the zebra finch (*Taeniopygia guttata*), the removal of the mother during postnatal development decreased GR and MR mRNA levels in the hypothalamus and MR mRNA levels in the hippocampus and cerebellum 19. However, our chosen model species is precocial, whereas the zebra finch is an altricial species. In precocial species, the HPA axis is functional from at least mid-incubation stage and precocial hatchlings show maximal HPA responsiveness at hatching 38,39. Chicken and mallard duck (*Anas platyrhynchos*) embryos have detectable endogenous CORT levels and can exhibit a stress response from the second half of incubation onwards 38,40,41. Conversely, in altricial species, it is assumed that there is very little prenatal functional development of the HPA axis and, indeed, several altricial species across a range of taxa exhibit a hyporesponsive HPA axis during early postnatal development 42–44. Consequently, it appears to be intuitive that the findings of the present study suggest that prenatal stress is the main developmental stage impacting on HPA axis regulation via modification of GR and MR receptor expression. One potential caveat of the present study is that we determined GR and MR mRNA levels but did not determine GR and MR protein expression in the studied regions. It has been suggested that mRNA and protein expression measures do not always correlate, as has been shown in the chicken 36; however, most studies that report both mRNA and protein expression do suggest good agreement between GR and MR mRNA and protein levels 45–51. For example, exposure to a single prolonged stress in the rat reduced GR and MR expression in terms of both protein and mRNA in the amygdala to the same extent 46. Moreover, the observed changes in GR and MR expression in the present study are in accordance with the mediation of the physiological acute stress response (see below). Therefore, we could expect to find the same modification at the post-transcriptional level because GR and MR mRNA and protein levels are usually coupled. However, it would be interesting to directly investigate whether GR and MR mRNA and protein levels are coupled in the Japanese quail.

Within the HPA axis, GR and MR play a crucial role in the regulation of baseline and stress-induced glucocorticoids and in the negative-feedback efficiency 10–12. Stress exposure during early life has been shown to affect these receptors expression in the brain. In mammals, both prenatal and postnatal stress exposures have been shown to decrease hippocampal MR and GR expression, as well as GR expression in the hypothalamus and in the pituitary gland. The decrease in both GR and MR mRNA in these different brain regions results in an altered HPA axis function through alteration of baseline or stress-induced glucocorticoid levels and/or negative-feedback during stress recovery 2,8,20,34,52,53. By contrast, some studies have shown that early-life stress exposure can result in an attenuated physiological stress response associated with an increased GR and/or MR expression within the HPA axis suggesting enhanced negative-feedback efficiency 25,35,54. In birds, studies investigating the consequences of early-life stress on GR and MR expression in the brain are limited and did not show consistent effects (see above). In the Japanese quail, we showed that prenatal stress increased the GR:MR ratio in the hippocampus, GR and MR expression in the hypothalamus, and GR expression in the pituitary gland. It has been suggested that an increase in the GR:MR ratio in the hippocampus should allow an increase in the effects of GR, resulting in a more efficient feedback and facilitating recovery from stress 11,17,55. Moreover, an increase in MR and GR expression in the hypothalamus, as well as in GR expression the anterior pituitary, should enhance HPA axis negative-feedback 4,8,10,11,13,16. Hence, prenatally stressed quail should exhibit a more efficient negative-feedback and, consequently, a more attenuated stress response to an acute stress. Indeed, it has been previously shown in these quail that prenatal stress exposure resulted in an attenuated acute stress response, with these birds showing a larger decrease in CORT level between 10 and 30 min after capture compared to control individuals 26. Therefore, modifications of both GR and MR expression within the HPA axis that we measured after exposure to prenatal stress can explain the attenuated physiological stress response observed in these quail via a more efficient negative-feedback loop.

We showed that MR expression in the hippocampus was decreased following postnatal stress in the form of unpredictable food availability and there was also a tendency for this decrease to occur after any of our experimental treatments. In the face of a stressful situation, hippocampal MR mediate the levels of circulating glucocorticoids, causing heightened evaluation of environmental information and the selection of the appropriate behavioural response 10,11,16,17. Reductions in MR mRNA expression in the hippocampus may be associated with a reduction of the HPA axis sensitivity 11,12,14,16. In agreement, in rats, the use of an MR antagonist reduced anxiety-like behaviours and increased exploration behaviour independent of GR expression levels 56,57. This suggests that a decreased expression of MR in the hippocampus may reduce the perception of a novel situation as stressful. In a previous study using the same quail, individuals exposed to only one early-life stress or to both stressors showed higher exploration and risk-taking behaviour in a novel environment 26. Individuals exposed to prenatal stress took less time to enter the novel environment and spent more time active in the novel environment. Individuals exposed to postnatal stress showed a lower latency to feed from unfamiliar and stressful enclosure. Finally, exposure to both early-life stresses had cumulative effects resulting in a lower latency to feed in the novel environment, an increased time spent away from the home cage and next to the stressful feeder, a higher probability of entering this stressful feeder and an increased access to food resources 26. Therefore, it is possible that the decrease in MR expression in the hippocampus may decrease the perception of the new environment as stressful and thus might partly mediate the increase in exploration and risk taking in these quail. However, because hippocampal MR expression reduction in two of these groups did not modify the physiological stress response, these behavioural changes were not directly mediated by a modification of glucocorticoid levels and another physiological mechanism should be involved. Further work is necessary to identify this physiological mechanism.

In the present study, we showed that exposure to pre- and postnatal stress resulted in long-term persistent changes in the expression of GR and MR receptors in different brain regions of the HPA axis in birds. These modifications of MR and GR expression within the HPA axis can explain the permanent programming of the HPA axis in prenatally stressed quail. They may also, at least partly, explain the programming of behaviour in quail exposed to prenatal and/or postnatal stress. Therefore, changes of gene expression in both receptors as a consequence of early-life stress exposure might comprise the mechanism that facilitates an adaptive response to later-life environmental conditions.
